# A novel gene, *ardD*, determines antirestriction activity of the non-conjugative transposon Tn*5053* and is located antisense within the *tniA* gene

**DOI:** 10.1111/1574-6968.12005

**Published:** 2012-10-03

**Authors:** Vladimir P Balabanov, Vera Yu Kotova, Gennady Y Kholodii, Sofia Z Mindlin, Gennadii B Zavilgelsky

**Affiliations:** 1 State Research Institute of Genetics and Selection of Industrial Microorganisms (GosNIIgenetika)Moscow, Russia; 2 Institute of Molecular Genetics, Russian Academy of SciencesMoscow, Russia

**Keywords:** restriction-modification system type I, antirestriction protein, mercury-resistance transposon, overlapping genes

## Abstract

The mercury-resistance transposon Tn*5053* inhibits restriction activity of the type I restriction-modification endonuclease EcoKI in *Escherichia coli* K12 cells. This is the first report of antirestriction activity of a non-conjugative transposon. The gene (*ardD*) coding for the antirestriction protein has been cloned. The *ardD* gene is located within the *tniA* gene, coding for transposase, on the complementary strand. The direction of transcription is opposite to transcription of the *tniA* gene.

## Introduction

Conjugative plasmids and conjugative transposons contain the *ardA*, *ardB* and *ardC* genes, coding for antirestriction proteins. The ArdA, ArdB and ArdC proteins specifically inhibit type I restriction-modification enzymes (Delver *et al*., 1991; Belogurov *et al*., 1993, 2000; Serfiotis-Mitsa *et al*., 2010). The ArdA proteins simultaneously inhibit restriction (endonuclease) and modification (methylase) acitivity of these enzymes (Delver *et al*., 1991; McMaahon *et al*., 2009), while the ArdB proteins inhibit only restriction activity of the enzymes (Belogurov *et al*., 1993; Serfiotis-Mitsa *et al*., 2010). These proteins differ considerably in both primary and tertiary structure. The ArdA proteins (165–170 amino acids) carry a considerable negative charge (−25: −30) and belong to the family of DNA mimic proteins, because their spatial structure is similar to the double-helical DNA in B form (McMaahon *et al*., 2009). The ArdB proteins (145–153 amino acids) usually carry a small negative charge (−1: −6) and form a structure of a compact tetraeder (Serfiotis-Mitsa *et al*., 2010*)*. The presence of the *ardA* and *ardB* genes helps mobile elements to overcome the restriction barriers, providing efficient ‘horizontal’ gene transfer between bacteria of various species and genera.

We have previously shown that the *merR* gene Tn*5053*, cloned in the vector pUC19 and introduced in *Escherichia coli* K12 strain JM83 shows an antirestriction effect against a type I restriction enzyme EcoKI. The presence of the *merR* gene in the cell increased the plating efficiency of the bacteriophage λ.0 with non-modified DNA about five- to seven-fold (Rastorguev *et al*., 1999). MerR is a transcriptional regulator of the *mer* operon. Here we demonstrate that the full-length mercury-resistance transposon Tn*5053*, when introduced in a bacterial cell within the vector pUC19, inhibits restriction activity of the EcoKI enzyme, decreasing it about 100-fold. We showed that a new gene, designated *ardD*, codes for a protein that shows antirestriction activity against EcoKI. This gene is located within the *tniA* gene (encoding transposase) on the complementary strand.

## Materials and methods

### Bacterial strains, bacteriophage, and plasmids

Relevant characteristics of the bacterial strains, bacteriophage and plasmids used in this study are described in Table 1. Routine cell growth was carried out at 37 °C in Luria–Bertani (LB) medium supplemented with antibiotics as appropriate.

**Table 1:** *Escherichia coli* strains and plasmids used in this study

Name	Genotype or description	Source or reference
Strain		
AB1157	F^-^ *thr-1*, *leu-6*, *proA2*, *his-4*, *thi-1*, *argE3*, *lacY1*, *galK2*, *ara14*, *xyl-5*, *mtl-1*, *tsx-*33, *rpsL*31, *supE44*, *r+m+*	N.E. Murray, UK
NK114	Δ*clpX::kan*, derivative of AB1157	N.E. Murray, UK
TG-1	*thi relA supE44 hsdR17 hsdM* Δ (*lac-proAB)* [F*′traD36 proAB lacIqZΔM15*]	VKPM ‘GosNIIgenetika’
MC1061	*araD139 Δ(araA-leu)7697 Δ lacX74 galK16 galE15 mcrA0 relA1 rpsL150 spoT1 mcrB1 hsdR2*	VKPM ‘GosNIIgenetika’
Plasmid		
pUC19	ColE1 origin, Amp^r^	Fermentas, Lithuania
pTZ57R	ColE1 origin, Amp^r^	Fermentas, Lithuania
pKLH53.1	Amp^r^, Hg^r^, pUC19 with Tn*5053* (8500 bp) of chromosome *Xanthomonas* sp. W17 inserted between PvuII/DraI and NdeI sites.	Kholodii *et al*. (1995)
pKLH53.1*tniQ1*	Deletion between the Acc651 and HpaI sites of pKLH53.2 inactivating *tniB* and *tniQ*	Kholodii *et al*. (1995)
pKLH53.1*tniQ2*	730-bp deletion between the ClaI and Acc651 sites within *tniQ* in pKLH53.1	Kholodii *et al*. (1995)
pKLH53.1*tniB2*	Insertion of the filled-in EcoRI fragment containing the Km^r^ cassette into the HpaI site within *tniB* of pKLH53.1	Kholodii *et al*. (1995)
pKLH53.1*tniA*	Insertion of the SalI fragment with a Km^r^ cassette from pUC4K into the SalI site within *tniA* of pKLH53.1	Kholodii *et al*. (1995)
pKLH53.2	*tni* operon Tn*5053* inserted in plasmid pACYC184	Kholodii *et al*. (1995)
pTL*HindIII-ClaI*	HindIII-ClaI fragment from the *mer* operon of Tn*5053* cloned in pUC19	This study
pTL2.5	HindIII fragment from the *mer* operon of Tn*5053* cloned in pUC19	This study
pTLΔ*HindIII*	Obtained by treatment of pKLH53.1 with HindIII and subsequent ligation	This study
pTLORF5	2300-bp KpnI SalI fragment from pKLH53.1 cloned in pUC19	This study
pSMΔORF5	Obtained by treatment of pTLORF5 with Eco47III and subsequent ligation	This study
pORF5	*orf-5* cloned in pUC19 under the *lac* promoter	This study
Bacteriophage λvir		R. Devoret, France

### Media and reagents

Luria–Bertani medium and LB agar (1.8% agar) were prepared according to Miller (1972). Antibiotics were added as required: ampicillin (100 μg mL^−1^), kanamycin (40 μg mL^−1^) and chloramphenicol (20 μg mL^−1^).

The enzymes for cloning were supplied by Fermentas.

### DNA isolation, restriction, ligation and transformation

Hybrid plasmids and vectors were isolated using a kit from Qiagen. Chromosomal DNA was isolated from the cells at late exponential phase of growth; the cells were lysed with lysozym and sodium dodecyl sulphate and the lysate was then treated with phenol with subsequent DNA sedimentation in ethanol.

Restriction, ligation of DNA fragments, electrophoresis in agarose gel, isolation of DNA fragments from the gel by electroelusion and transformation of calcium cells were performed in *E. coli* as described (Sambrook *et al*., 1989).

### Construction of recombinant plasmids

The plasmid pTLΔ*HindIII* was obtained by treatment of pKLH53.1 with HindIII and subsequent ligation. The HindIII fragment of 2.5 kbp and HindIII-ClaI fragment from the *mer* operon of Tn*5053* were cloned in pUC19 under the *lac* promoter: pTL2.5 (2.5-kbp HindIII fragment) and pTL*HindIII-ClaI* (HindIII-ClaI fragment). The fragment *tniA,B,Q* Tn*5053* (2.3 kbp) was cloned in pUC19 under the *lac* promoter (pTLORF-5). Hybrid plasmid pSMΔORF-5 was obtained by eliminating the DNA between the Eco47III sites within the *orf-5* gene in pTLORF-5 (see Fig.). In pORF-5, a 483-bp fragment from the *tniA* gene was cloned in pUC19 under the *lac* promoter (see Fig.). The DNA fragment containing the gene *orf-*5 was amplified by PCR using the following primers: Tn5053dir, 5′-GCAGAGGGTGACGGCCGGATGG-3′; Tn5053rev, 5′-CACGGCGATGCAGATGATCCACG-3′ and plasmid pKLH53.1 DNA as a template. Amplification was carried out at the conditions recommended by the manufacturer. The amplification product was purified by electrophoresis and cloned in T-vector pTZ57R. A 483-bp fragment was then recloned into pUC19 at XbaI and BamHI restriction sites to construct pORF-5. For the other plasmid constructs of the pKLH series see Kholodii *et al*. (1995).

### Estimation of antirestriction activity

The antirestriction activity of plasmid was defined as the efficiency of plating (EOP) of unmodified phage λ.0 on the experimental (plasmid-bearing) strain divided by the EOP on the plasmidless restricting strain (Delver *et al*., 1991). The EOP (in Table 2 designated К) was calculated as: phage titre on the restricting strain (NK114)/phage titre on a nonrestricting strain (TG-1). Unmodified phages, denoted by λ.0, were grown on *E. coli* TG-1 r^−^m^−^, which lost restriction and modification functions. All assays were performed in triplicate and at least 50 phage plaques per plate per experiment were counted. Experiments were performed on numerous days with fresh samples and control experiments performed each day. Little variation was observed during the replicate experiments. The standard deviation for the antirestriction results is 25% or less.

**Table 2:** Comparison of antirestriction activity of cloned fragments and deletion and insertion mutants of the transposon Tn*5053*

Plasmid	Coefficient of restriction (*K*)*	Restriction relief (*R*)†
pUC19 (control)	1.0 × 10^−5^‡	1
pKLH53.1	1.1 × 10^−3^	110
pKLH53.1*tniA*	1.0 × 10^−3^	100
pKLH53.1*tniB2*	9.5 × 10^−4^	95
pKLH53.1*tniQ2*	1.2 × 10^−3^	120
pKLH53.1*tniQ1*	9.6 × 10^−4^	96
pTLΔ*Hin*dIII	1.0 × 10^−5^	1
pTL*Hin*dIII-*Cla*I	1.0 × 10^−5^	1
pTL2.5	1.0 × 10^−5^	1
pKLH53.2	1.0 × 10^−5^	1
pTLORF-5	1.1 × 10^−3^	110
pSMΔORF-5	1.0 × 10^−5^	1
pORF-5	5.3 × 10^−3^	530

* The coefficient of restriction (*K*) was determined as the ratio of the titre of phage λ.0 on strain NK114 r^+^m^+^ to the titre of the same phage on strain TG-1 r^−^m^−^.

† The restriction relief factor *R* = *K*_+_/*K*_−_, where *K*_+_ is *K* for NK114 with a plasmid, and *K*_−_ is *K* for NK114 without a plasmid.

‡ Mean of three independent experiments.

## Results

### Antirestriction activity of the transposon Tn*5053*, its deletion and insertion mutants

#### Plasmids with antirestriction activity

**Fig 1:**
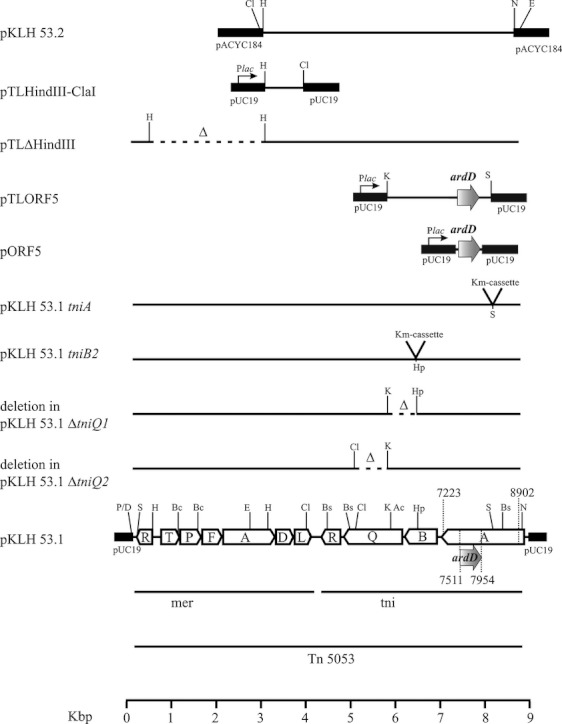
Structure of pKLH53.1, subcloned fragments, insertion and deletion mutants. EV, EcoRV; Bc, BclI; Bs, BssHII; Cl, ClaI; D, DraI; E, EcoRI; Ac, Acc65I; H, HindIII; Hp, HpaI; K, KpnI; N, NdeI; P, PvuII; S, SalI.

Data on antirestriction activity of the recombinant plasmid pKLH53.1, containing Tn*5053*, are given in Table. The factor of restriction relief (*R*) is about 100. We suspected that the nucleotide sequence of the mercury-resistance transposon Tn*5053* contains a fragment encoding an antirestriction protein. We used both insertion and deletion mutants of Tn*5053* for all transposition genes (*tni*) as well as plasmid constructs containing various fragments of the Tn*5053* DNA, while searching for the locus responsible for the antirestriction activity (Fig. 1). The results of searches for the determinant of antirestriction activity within Tn*5053* are shown in Table 2. It is evident that neither insertion (plasmids pKLH53.1*tniA*, pKLH53.1*tniB2*) or deletion (plasmids pKLH53.1*tniQ2* and pKLH53.1*tniQ1*) mutations of the *tni* genes have any effect on antirestriction activity: about 100-fold decrease in EcoKI restriction level is preserved.

#### Plasmids without antirestriction activity

Deletion of the major part of the *mer* operon (plasmid pTLΔ*Hind*III) completely removed the effect of antirestriction (Table). We assumed that the location of the gene coding for an antirestriction protein is within the *mer* operon. However, the recombinant plasmids pTL*Hin*dIII-*Cla*I and pTL2.5 with fragments HindIII-ClaI and HindIII from the *mer* operon (without the *merR* gene) in vector pUC19 show no antirestriction effect (Table 2). No antirestriction effect was also observed for the hybrid plasmid pKLH53.2, containing all the genes *tni* Tn*5053* under its own promoter (in vector pACYC184; Fig., Table 2). A paradox appeared: the *mer* operon together with the transposition genes (*tni*) of Tn*5053* produce an antirestriction effect, while the plasmids with separately cloned *mer* operon or *tni* genes show no antirestriction effect.

### Construction of recombinant plasmids containing *orf-5* and evaluation of their antirestriction activity

**Fig 2:**
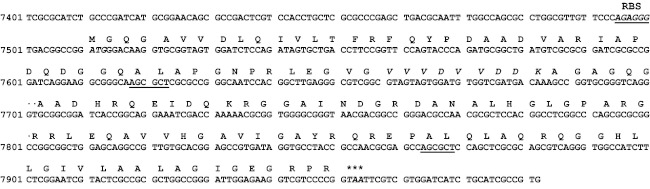
Nucleotide sequence of the gene *orf-5* (*ardD*) and amino acid sequence of its product (147 amino acids), encoded by the complementary strand of the gene *tniA* in transposon Tn*5053*. RBS is shown in italics and underlined, a putative antirestriction motif is shown in italics and Eco47III sites are underlined.

We considered that the nucleotide sequence coding for the ORF with antirestriction activity is located within the region of the *tni* genes, but orientated in reverse to the direction of transcription of the *tni* genes. Consequently, the coding strand for this ORF is the same as for the *mer* operon. If so, transcription of this DNA fragment passes from the side of the *mer* operon. We analysed the DNA sequence from the region of the *tni* genes of Tn*5053* in reverse direction, and found several *orf*s. Of main interest was *orf-5*, encoding a negatively charged protein with a motif close to the antirestriction motif of the proteins Ard (Fig. 2). The protein ORF-5 contains 147 amino acid residues of summary charge −1. It is encoded by *orf-5* at positions 7511–7954 on the complementary strand of the *tni*A gene (positions numbered according to the nucleotide sequence of Tn*5053*, deposited in DBJ/EMBL/GenBank under accession number L40585). The nucleotide sequence located upstream of the initiation codon (AGAGGGT) is virtually identical to the canonical ribosome binding site (RBS) sequence (AGGAGGT). Note that other ORFs found along the complementary strand in the region of the genes *tni* Tn*5053* do not contain RBS sequence upstream of the initiation codon.

To test the hypothesis of antirestriction activity of *orf-5*, we constructed a hybrid plasmid using the 2300-bp KpnI-SalI DNA fragment from *orf-5* containing region *tniA,B,Q*. This fragment was cloned under the *lac* promoter in vector pUC18 (pTLORF-5, Fig. 2). Introduction of this plasmid into cells of strain NK114 produced an antirestriction effect similar to that observed for the wild-type Tn*5053*, about 100-fold (Table 2). Internal deletion in the *orf-5* gene was produced by Eco47III restriction endonuclease treatment of pTLORF-5. In the resulting plasmid pSMΔORF-5, a major part of *orf-*5 (245 bp; nucleotides 7621–7866 in the L40585 sequence) was deleted, including the putative antirestriction motif VVDVVDDKA (Fig. 2). The antirestriction effect in *E. coli* NK114 cells, containing pSMΔORF-5, disappeared completely (Table 2). For further evaluation of the role of *orf-5* in this antirestriction effect, we amplified *orf-5* together with the RBS and cloned them in pUC19 under the *lac* promoter (for details see Materials and methods). After the plasmid obtained (pORF-5) was introduced into NK114 cells, the antirectriction factor R was estimated. Plasmid pORF-5 showed a considerable antirestriction effect: efficiency of the λ.0 phage plating was about 500-fold higher than the control level (cells with pUC19) (Table 2).

## Discussion

It has been shown that the genes encoding the antirestriction proteins (ArdA, ArdB, ArdC) may be located within conjugative plasmids and conjugative transposons (Delver *et al*., 1991; Belogurov *et al*., 1993, 2000; McMaahon *et al*., 2009; Serfiotis-Mitsa *et al*., 2010). Here we show for the first time that a similar gene is also present within a non-conjugative transposon (Tn*5053*). Analysis of the deduced amino acid sequence of ORF-5 revealed that this protein has no similarities to the known Ard proteins (ArdA, ArdB and ArdC types) except the ‘antirestriction’ motif conserved for all known Ard proteins. This suggests that ORF-5 may be classified as a new type of Ard protein, which we designate ArdD. The N-terminal region of ArdD has a high degree of similarity (about 39% identity and 53% similarity) to the region of the MerR protein (312–367 amino acids) of *Desulfovibrio vulgaris* strain ‘Miyazaki F’ (NCBI reference sequence YP_002436545.1; Fig.). Interestingly, the total negative charge of homologous sequences ArdD and MerR is virtually the same, −5 and −7, respectively. The location of the *ardD* gene appears to be unusual: inside a transposition gene (*tni*A) with transcription at the complementary strand (Fig. 1). Overlapping genes in bacterial genomes are rare. For example, most strains of *Shigella flexneri* 2a and enteroaggregative *E. coli* carry a highly conserved chromosomal locus which encodes a 109-kDa secreted mucinase Pic and, on the opposite strand in overlapping fashion, an oligomeric enterotoxin ShET1, encoded by the *setA* and *setB* genes. The *setB* gene is transcribed from a promoter which lies more than 1.5 kb upstream of the *setB* gene (Behrens *et al*., 2002). According to our data, the *ardD* gene promoter is also located distantly from the *ardD* gene in the region of the *mer* operon, at a distance of more than 3 kbp. We suggest that other non-conjugative transposons may also contain genes that encode products that can inhibit the restriction endonucleases, thereby efficient overcoming restriction barriers. Note that the *tniA* gene is usually present in integrons and composite transposons conferring antibiotic resistance and is widely distributed among environmental and clinical bacteria. As an example, the transposon Tn*6006* contains a nucleotide sequence identical to *ardD* in the *tniA* gene. The Tn*6006* transposon belongs to the group of recombinant transposons containing integrons (Fluit & Schmitz, 1999; Labbate *et al*., 2008).

**Fig 3:**
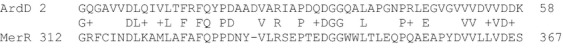
Comparison of the amino acid sequences of ArdD (Tn5053) and MerR (*Desulfovibrio vulgaris* strain ‘Miyazaki F’). Aligment was done with the program NCBI blast. ArdD (2–58 amino acids) and MerR (312–367 amino acids) homologous regions: identity = 39%, similarity = 53%.
